# Community Organization Membership, Financial Security, and Social Protection among Female Sex Workers in India

**DOI:** 10.1177/2325958218811640

**Published:** 2018-11-16

**Authors:** Sangram Kishor Patel, Anrudh Jain, Madhusudana Battala, Bidhubhusan Mahapatra, Niranjan Saggurti

**Affiliations:** 1 HIV and AIDS Program, Population Council, New Delhi, India; 2 Population Council, New York, NY, USA

**Keywords:** community organization membership, financial security, social protection, HIV, India

## Abstract

The purpose of this study is to examine the female sex workers’ (FSWs) community organization (CO) membership, their financial and social protection security, and the relationship between these factors among FSWs in India. Data from 4098 FSWs collected under the Avahan-III baseline evaluation survey—2015 in 5 high HIV prevalence states (Maharashtra, Tamil Nadu, Karnataka, Telangana, and Andhra Pradesh) in India were used here. More than three-fifths (77%) were registered CO members, of whom 79% had been CO members for more than 1 year. The likelihood of having high financial security (19% versus 10%; adjusted odds ratio [AOR]: 1.7; 95% confidence interval [CI]: 1.3-2.1) and social protection security (13% versus 6%; AOR: 1.6; 95% CI: 1.2-2.0) was 2 times higher among FSWs who were CO members compared to those who were not. The study offers important insights into furthering CO membership to address financial and social vulnerability as a path to a sustainable reduction of HIV risk.

## Introduction

• **What do we already know about this topic?**
  Community Organizations provide a platform to key population in bringing them together and demand for basic rights and services. Collectivization helps key population in accessing quality health and social welfare services and adopting safe sex behavior.
• **How does your research contribute to the field?**
  This study examined whether membership in community-led organizations helped FSWs in improve their financial and social security. In addition, this study also assessed the relationships between duration of CO membership and frequency of CO members’ interactions with COs with FSWs’ financial security and social protection.
• **What are your research’s implications toward theory, practice, or policy?**
  Being member of a CO is critical to address financial and social vulnerability as well as sustain the reduction in HIV risk among key populations. Efforts are required to strengthen current community advocacy and engagement systems so that COs can continue to work on addressing vulnerabilities faced by marginalized populations.

Over the years, community-led interventions have been proven to be instrumental in HIV prevention among marginalized populations with increased risk of HIV across the world.^1–4^ This strategy encourages collectivization to bring about structural change and aims not only to empower marginalized key populations, particularly female sex workers (FSWs) and men who have sex with men (MSM) as a group for vulnerability reduction, but also increasingly allows them to make decisions and shape their own lives, which in turn influences the adoption and maintenance of low-risk behaviors.^5–7^ Evidence shows that community-based groups or community organizations (COs) are essential to the global response to the AIDS pandemic, particularly in low- and middle-income countries.^8,9^ Community organizations can be defined here as formally organized communities through institutions set up and led by a particular community (eg, FSWs, MSM), and they can range from small self-help groups to large trade unions.^10,11^

Previous literature has argued that COs are influential in working at the grassroots level, in organizing outreach activities among marginalized populations to tackle poverty and disease (eg, HIV/AIDS), and in providing care, support, and treatment services.^12–15^ Further, COs also engage local communities through other programs, such as income-generating activities, cash transfers, and microcredit schemes, especially those targeting marginalized groups with limited access to services and financial resources.^15,16^ Community organization membership affects the formation of self-identity as well as a person’s attitude toward others.^17^ It is also associated with lower rates of HIV prevalence among marginalized populations, especially FSWs,^18–20^ and with a number of factors linked to lower risk of acquiring HIV, including alcohol consumption,^18^ intimate partner violence,^20^ and sexual behavior.^21^ Community organizations membership can provide FSWs the social-economic and psychological support to “reconstrue” threats to one’s sense of identity and well-being.^17,22,23^

Researches established that engagement with community groups can result in accessing the basic health services and reducing the HIV vulnerabilities among key populations.^24–26^ In addition, continued support of CO staff, duration of association with CO, and group interaction with other members could result in improvements in autonomy, self-confidence, and self-efficacy,^15,23^ which ultimately promotes sustained behaviors. However, an examination of the link between CO membership and vulnerability reduction in the context of financial security and social protection among FSWs has not been adequately investigated. This study is designed based on a theory of change that describes the pathways through which CO membership can address FSW vulnerability (in terms of social and financial security in this context). The theory of change hypothesized that FSWs’ membership in community-led organization enables them to gain knowledge about schemes, which includes knowing the benefits associated with and process of availing various schemes. The COs play the role of facilitator by providing a unified help desk to process the application to apply for different social protection and financial schemes. A little has been explored about the relative benefits of CO membership, duration of CO membership, and frequency of interactions with CO in the Indian context. In this regard, this study’s main aim is to examine the FSWs’ CO membership, their financial security and social protection, and the relationship between these factors in India. Additionally, the current study has examined the relationships between duration of CO membership and frequency of CO members’ interactions with COs with FSWs’ financial security and social protection.

## Materials and Methods

### Avahan Phase-III Program

The India AIDS initiative of Bill and Melinda Gates, with a focused ambition to prevent transmission of HIV among key population across 6 high prevalence states in India, was launched in 2003^27^ and paved its way through 3 phases. The Avahan-III program (2014-17) is the last and final phase of intervention where the focus was on building capacity of COs to enhance their skills in program management and resource mobilization as well as to address individual vulnerabilities of key population to sustain their safe-sex behaviors. The phase III of Avahan program is implemented across 5 states in India, that is, Andhra Pradesh, Telangana, Tamil Nadu, Karnataka, and Maharashtra. The program laid its foundation to address vulnerability of the key populations through 4 crucial pillars—financial security, social protection, crisis response at individual level, and institutional development at CO level. The pillars were designed to provide organized support to FSWs to obtain their social and economic entitlements, such as ration cards, Aadhaar card, bank accounts, voter IDs, land *patta*, gas connections in addition to benefits from social protection schemes. A unified help desk was set up in each CO to facilitate FSWs to access to different social and economic services. Each COs had about 4 to 5 field workers (FWs) who oversaw the functioning of unified help desk per 250 to 300 key populations. The allocation of the number of key population to one FW is done purely based on the geographical contiguity.

### Study Settings and Design

This study used the data from the Avahan-III baseline evaluation survey, collected during August to November 2015 in 5 high HIV prevalence states of India—Andhra Pradesh, Telangana, Tamil Nadu, Karnataka, and Maharashtra. The baseline survey collected information on different vulnerabilities and key behavioral indicators (eg, HIV risk behaviors, social protection, financial security, violence, institutional development.). At the time of the baseline survey, Avahan-III program was being implemented across 87 COs, in which 75 COs were focused with FSW populations and 12 COs with MSM and transgender populations.

### Sampling and Sample Size

In the Avahan-III baseline survey—2015, sample size and power considerations were constructed based on our primary outcomes of interest: savings through formal sources, consistent condom use, and proportion who experienced any violence. The sample size calculation assumed a 2-sided χ^2^ test with continuity correction and a significance level of 0.05. We estimated the intraclass correlation of 0.15 and a nonresponse rate of 33% (based on previous surveys).^28^ With this we calculated a sample size of 900 from 10 COs per state to present state-level estimates. Given that Telangana had only 7 COs, it was decided to consider the state of Andhra Pradesh and Telangana as one entity for the study purpose. It has to be noted Andhra Pradesh and Telangana were considered as one state prior to June 2014. The estimated sample size at state level translated to 90 interviews per CO. The sample size at CO level was rounded off to 100, which resulted in 1000 interviews at the state level.

A 3-stage cluster sampling method was used with selection of CO in the first stage, clusters in the second stage, and respondents in the third stage. In each state, 10 COs were randomly selected, except for Tamil Nadu where all 9 COs implementing the program were selected by default. The catchment area covered under one FW was considered as a cluster. Three such clusters were randomly selected from each selected CO. The program implementing agency undertook a detailed mapping exercise covering all key populations in the program coverage area using a Membership Engagement Communication Tool. The tool collected basic information on their demographics, place of solicitation, and membership status with a CO for all key populations. This list was considered as the sampling frame for selection of FSWs within FW areas. About 100 respondents were selected within each selected cluster. At the end of the survey, a total of 4098 FSWs were interviewed.

### Data Collection

The interviews were conducted by trained female investigators with verbal and written skills in the local language of each state. After developing the structured questionnaire for FSWs in English, it was translated into the local languages. Prior to the survey, pretesting of questionnaire was done in similar community groups. To ensure privacy, all the interviews were held in a private location specifically hired for the survey or in a location convenient to the study participants.^28^ To maintain accuracy and completeness of the filled-in questionnaires, the trained field staff checked the data instantly after the interview. Data were entered in Census and Survey Processing System (CSPro) (version 5.0) by trained data entry officers.

### Ethics Statement

The consent processes, questionnaires, and study design were reviewed and approved by the institutional review board of Population Council and Sahara’s Centre for Residential Care and Rehabilitation. In accordance with the protocol, written consent was obtained from respondents who can read and sign prior to their participation in the survey and steps were taken to ensure confidentiality. Verbal consent was taken from respondents who could not read or write. The verbal consent was taken in the presence of a coparticipant of respondent’s choice or an individual from the program implementing organization (ie, Swasti). For ethical reasons, FSWs aged 18 years or above were part of the sampling frame, and among them, information on selected participants was collected accordingly. No names and information were recorded on the questionnaires. Compensation was provided for travel expenses to the participants in the study, at the same time they were referred to local CO project sites run by the implementing partner organization for more information and services. All the respondents were given the contact details of lead researchers from the Population Council to communicate their concerns on the research and to get more information about study.^28^

### Measures

The measures and sociodemographic variables used in this study were selected as per the review of literature, theoretical consideration, objectives of the article, and availability of variables in the data set. The sociodemographic variables considered in the analysis were age (≤30 years, >30 years), education (illiterate, literate), marital status (never married, currently married, and others [widowed/divorced/separated/deserted]), place of solicitation for sex work (home, brothel/lodge/hotel, public places/street/highways, others), living status (living with family members/husband, living alone, and living with others), community membership (no, yes, community membership has been defined here as FSWs who were registered with a CO at the time of the survey), duration of membership (≤1 year and >1 year), and frequency of interaction with the CO among the members (not daily, daily).

#### Financial security

Financial security of FSWs was measured by a composite index “Financial Security Intervention Coverage Index (FSICI)” calculated by using 4 components of financial security: (1) having a savings account in a bank or post office, (2) having invested in saving products (such as recurring deposits, fixed deposits, public provident fund, national savings certificates, co-op, shares, mutual fund), (3) invested in insurance (life, health, and accident), and (4) other investments (gold, land, business). Scores were assigned as per their importance for financial security and discussions between technical experts and the program implementation agency (eg, FSWs, having a saving account [no] and having any saving product/insurance/investment [no] was assigned a score of 1; having a saving account [no] and having any saving product/insurance/investment [yes] was assigned a score of 2; having a saving account [yes] and having any saving product/insurance/investment [yes] was assigned a score of 3; having a saving account [yes] and having any saving product/insurance/investment [yes] was assigned a score of 4; having a saving account [yes] and having any saving product/insurance/investment [yes] was assigned a score of 5). The validation of the FSICI index was already done with program data of the implementation agency. The total FSICI scores ranged from 1 (minimum) to 5 (maximum). The composite scores were further divided into 2 categories, low (<3 scores: having low financial security) and high (≥3 scores: having high financial security) based on the mid-value of the total score.

#### Social protection

Social protection among FSWs was measured by a composite index “Social Protection Intervention Coverage Index (SPICI)” using 2 components: (1) number of civic identities FSWs possess (eg, Aadhaar card, Voter ID, Ration cards, PAN card, etc) and (2) number of social protection schemes (eg India Awas Yojna, Child education scheme, pension scheme, health scheme, etc.) from which FSWs have benefited. The scores were assigned as per their importance for social protection and discussions between technical experts and the program implementation agency (eg FSWs, having ≥1 civic identity and benefited from any social protection scheme [0] was assigned a score of 1; having >1 civic identity and benefited from any social protection scheme [0] was assigned a score of 2; having ≥1 civic identity and benefited from any social protection scheme [1] was assigned a score of 3; having ≥1 civic identity and benefited from any social protection scheme [2] was assigned a score of 4; having ≥1 civic identity and benefited from any social protection scheme [>2] was assigned a score of 5). The validation of the SPICI index was already done with program data of the implementation agency. The total SPICI scores ranged from 1 (minimum) to 5 (maximum). The composite scores were further divided into 2 categories: low (<3 scores: low social protection) and high (≥3 scores: high social protection) based on the mid-value of the total score.

### Data Analysis

Descriptive statistics (ie, means, standard deviations, and proportions) and bivariate analyses were conducted to examine the strength and association of sociodemographic characteristics, community membership, financial security, and social protection among FSWs. The respective *P* values for the bivariate analysis were calculated through χ^2^ test, and Fisher exact test was used in the case of FSICI for small samples. To assess the relationships of the degree of financial security and social protection with CO membership, adjusted odds ratios (AORs) and their 95% confidence intervals (CIs) were calculated through multiple logistic regression, adjusting for sociodemographic characteristics. For each dependent variable, we fitted 3 separate logistic regression models: one for overall sample with membership in CO as the key predictor variable; second and third for only for those having CO membership with duration of membership and frequency of interaction as key predictors. All analyses were conducted using STATA software (version 13.2).

## Results

Of 4098 FSWs, over half of the FSWs included in the study were older than 30 years (66.5%), currently married (62.1%), literate (58%), and living with family members/husband (60%; Table 1). More than three quarters of the FSWs (77.1%) were registered CO members. Of these, 79% had been CO members for more than 1 year and 3% were having daily interaction with CO staff. The characteristics of FSWs (such as age, education, marital status, place of solicitation, and living status) differed significantly by their CO membership status. Based on the FSICI, a little less than one-fifth of FSWs (17%) had high financial security; high financial security was higher among FSWs who were members of COs than nonmembers of COs (19% versus 10%, *P* < .001; Table 2). Similarly, based on SPICI, one-tenth of FSWs (11%) had high social protection; high social protection was higher among FSWs who were members of COs compared to nonmembers of COs (13% versus 6%, *P* < .001). The in-depth analysis shows that age, marital status, and place of solicitation were significantly associated with both FSICI and SPICI of FSWs.

**Table 1.** Sociodemographic Characteristics of Female Sex Workers by Community Organization Membership in India, 2015.^a^

Sociodemographic Characteristics	% (n) or Mean (SD)	Community Organization Membership		*P* Value
		No	Yes	
Age, mean (SD)	34.4 (6.6)			
Age				<.001
≤30 years	33.5 (1374)	46.7 (438)	29.6 (936)	
>30 years	66.5 (2724)	53.3 (500)	70.4 (2224)	
Education				.053
Illiterate	42.5 (1740)	45.2 (424)	41.7 (1316)	
Literate	57.5 (2358)	54.8 (514)	58.4 (1844)	
Marital status				<.001
Currently married	62.1 (2545)	63.0 (591)	61.8 (1954)	
Never married	7.7 (314)	17.8 (167)	4.7 (147)	
Others^b^	30.2 (1239)	19.2 (180)	33.5 (1059)	
Place of solicitation				<.001
Home based	25.1 (1030)	12.6 (118)	28.9 (912)	
Lodge/brothel based	29.9 (1227)	58.7 (551)	21.4 (676)	
Street/public places	21.6 (887)	18.7 (175)	22.5 (712)	
Others^c^	23.3 (954)	10.0 (94)	27.2 (860)	
Living status				<.001
Living with family members/husband	59.9 (2453)	55.5 (521)	61.1 (1932)	
Living alone	21.8 (895)	17.2 (161)	23.2 (734)	
Living with others^d^	18.3 (750)	27.3 (256)	15.6 (494)	
Member of CO				
No	22.9 (938)			
Yes	77.1 (3160)			
Duration of membership (N = 3160)				
≤1 year	-	-	20.7 (655)	
>1 year	-	-	79.3 (2505)	
Frequency of interaction with CO (N = 3160)				
More than a week	-	-	60.8 (1923)	
Weekly	-	-	36.1 (1142)	
Daily	-	-	3.0 (095)	
Total	100.0 (4098)	100.0 (938)	100.0 (3160)	

^a^ *P* values were calculated through χ^2^ test.

^b^ Includes divorced/separated/deserted.

^c^ Others include rented room/massage parlor/others.

^d^ Others include living with madam/sex workers/partner.

**Table 2.** Financial Security and Social Protection by Community Organization Membership among FSWs in India, 2015.^a^

Financial Security	% (n)/Mean (SD)	Community Organization Membership		*P* Value
		No	Yes	
Having a savings account in a bank or post office	68.5 (2804)	51.7 (483)	73.5 (2321)	<.001
Having saving products	4.3 (174)	5.0 (47)	4.0 (127)	.186
Having insurance (life, health, and accident)	13.1 (537)	3.9 (37)	15.8 (500)	<.001
Having other investments (gold, land, business)	4.3 (177)	4.8 (45)	4.2 (132)	.412
Financial Security Intervention Coverage Index (FSICI)				<.001
1	29.5 (1207)	46.7 (437)	24.4 (770)	
2	53.8 (2203)	43.1 (403)	57.0 (1800)	
3	13.9 (568)	8.2 (77)	15.5 (491)	
4	1.5 (63)	1.3 (12)	1.6 (50)	
5	1.3 (57)	0.6 (06)	1.5 (48)	
Degree of FSICI				<.001
Low (<3)	83.3 (3410)	89.8 (840)	81.3 (2570)	
High (≥3)	16.7 (684)	10.2 (95)	18.7 (589)	
Social protection				
Average number of civic identities FSWs possess	3.27 (1.26)	2.84 (1.41)	3.40 (1.18)	<.001
Average number of social protection schemes from which FSWs have benefited	0.13 (0.38)	0.07 (0.27)	0.15 (1.40)	<.001
Social Protection Intervention Coverage Index (SPICI)				<.001
1	9.6 (383)	18.7 (175)	6.6 (208)	
2	79.3 (3250)	75.4 (707)	80.5 (2543)	
3	9.9 (405)	5.5 (52)	11.2 (353)	
4	1.4 (58)	0.3 (03)	1.7 (55)	
5	0.1 (02)	0.1 (01)	0.03 (01)	
Degree of SPICI				<.001
Low (<3)	88.7 (3633)	94.0 (882)	87.1 (2751)	
High (≥3)	11.4 (465)	6.0 (56)	12.9 (409)	
Total	100.0 (4098)	100.0 (938)	100.0 (3160)	

Abbreviation: FSW, female sex worker.

^a^ *P* values were calculated through χ^2^ test, Fisher exact test for SPICI, and *t* test.

The multivariate analysis confirms the results from bivariate analysis (Table 3). Results show that the likelihood of having financial security (AOR: 1.7; 95% CI: 1.3-2.1) and social protection (AOR: 1.6; 95% CI: 1.2-2.0) were nearly 2 times higher among FSWs who were members of COs compared to those who were nonmembers of a CO. Further, the odds of reporting financial security were significantly higher among FSWs (AOR: 1.2; 95% CI: 1.1-1.6) who were CO members for more than 1 year compared to others. Female sex workers who were interacting with COs daily were nearly 2 times more likely to be financially secure (AOR: 1.6; 95% CI: 1.1-2.4) than those who were not interacting daily.

**Table 3.** Relationship Between Community Organization Membership with Degree of FSICI and SPICI among FSWs in India, 2015.^a^

	Degree of Financial Security Intervention Coverage Index (FSICI)		Degree of Social Protection Intervention Coverage Index (SPICI)	
	% (n)	AORs (95% CIs)	% (n)	AORs (95% CIs)
Community organization membership (N = 4098)				
No	10.2 (95)	Reference	6.0 (56)	Reference
Yes	18.7 (589)	1.7 (1.3-2.1)^b^	13.0 (409)	1.6 (1.2-2.0)^b^
Duration of membership (N = 3160)				
≤1 year	15.1 (99)	Reference	11.8 (77)	Reference
>1 year	19.6 (490)	1.2 (1.1-1.6)^c^	13.3 (332)	1.1 (0.8-1.4)
Frequency of interaction with CO among members (N = 3160)				
Not daily	18.1 (564)	Reference	12.9 (394)	Reference
Daily	26.3 (25)	1.6 (1.1-2.4)^c^	15.8 (15)	1.2 (0.7-1.9)

Abbreviations: AORs, adjusted odds ratios; CI, confidence interval.

^a^ AORs were adjusted for age, education, marital status, living arrangements, place of solicitation, and state.

^b^ Values significant at 1% level of significance.

^c^ Values significant at 5% level of significance.

## Discussion

The findings of the study establish a significant association between CO membership and financial security and social protection among FSWs. Previous studies also corroborate that community group membership has a positive influence on reducing key populations’ vulnerabilities (including financial and social vulnerabilities).^23,24,26,29^ Community organizations have emerged as a facilitating source for FSWs, which they can rely on for care and support; at the same time CO membership has been helping them to be financially and socially secure. The communities identified basic civic identity cards, social protection schemes along with financial services (eg, savings accounts, savings on products, insurance schemes, and investments) as the most needed social and financial benefit. This preference can perhaps be attributed to poverty and the community’s immediate need for essential commodities as well as to the COs’ program outreach and advocacy for these entities. Community organizations acts a social bridge of interactions between FSWs and government departments, banks, post offices, and other civil societies. Facilitating such interactions and establishing communications between these groups have been an essential step to make the government officials recognize social, cultural, political, and legal obstacle that FSWs face in their lives.^24^

Previous studies have highlighted social capital as a helpful measure in HIV prevention.^18–20^ Studies from South Africa and India have shown that social capital is associated with less stigmatizing attitudes toward marginalized populations and better provision of services.^30,31^ These findings are important in the context of an understanding of how CO membership can reduce stigma, which could be helpful in identifying strategies for strengthening and expanding their role in facilitating access to basic social and financial services. In addition, McMillan and Chavis’s sense of community theory supports this and states that an association with community or community membership has always been beneficial for people, as it works through 5 attributes: boundaries, emotional safety, a sense of belonging and identification, personal investment, and a common symbol system. These attributes always work together, which contribute to the welfare of the people.^32^

The study findings further validate a significant association between FSWs’ high financial security and duration of CO membership; FSWs who have a longer duration of association or membership with COs have higher financial security compared to their counterparts. The findings are similar to other studies conducted among women from marginalized groups.^25,29^ In Kenya, Walton, et al found that community group membership and duration of membership are positively associated with income, food security, and strengthening livelihood assets, which potentially impact additional outcomes (well-being, reduce vulnerability, and more sustainable land use) among women farmers.^25^ However, the study did not show a significant relationship between the duration of membership and social protection. This study further added to the literature that the likelihood of having high financial security among FSWs is significantly associated with frequency of interaction with CO. The findings show that although frequency of daily interactions of FSWs with CO was low, it has a significant and positive association with reduced vulnerabilities (particularly in terms of financial security). It can be stated here that the frequency of daily interactions was low as the program was in the very nascent stage and which may be increased as program outreach paces up or widens.

The study shows that degree of financial security (15% versus 20%) and social protection (12% versus 13%) is low among FSWs those who are members of COs for the last 1 year compared to the FSWs who are members of COs for more than 1 year. The gain in availing the financial security and social protection services among FSWs under the Avahan-III program in the last 1 year is substantially high compared to the gain prior to Avahan-III program (>1 year), which is the combined gain from earlier phases of program. Hence, we believe that strengthening of financial security and social protection services among CO members has shown an aspiring result within a year of the program implementation and the trend will be continued as Avahan-III program will be intensified.

Although the study findings offer several important insights on CO membership and social and financial security, they must be cautiously interpreted with certain limitations. The financial and social security indicators are based on self-reports which may be susceptible to social desirability and recall biases. The analysis is cross-sectional and causality cannot be assumed as in the case of prospective research studies. Further research is needed to assess whether CO membership reduces FSWs’ financial and social vulnerabilities. Furthermore, the study findings are only representative to the FSWs of the intervention areas, hence cannot be generalized to the whole FSW community. The other limitation with the analysis is the lack of sampling weight to account for the heterogeneity in size of population between states. In order to compensate the bias, all the multivariate analysis has been controlled for state. Finally, although this study establishes a significant positive association between CO membership and social and financial security among marginalized communities in terms of increasing FSWs’ access to social entitlements, financial services, and social protection schemes at the baseline, a longitudinal analysis on this topic will be useful and add to the literature. Future research is required to explore the relationship between these dimensions using the longitudinal data.

In conclusion, the study findings offer important insights into furthering CO membership to address financial and social vulnerability as a path to a sustainable reduction of HIV risk. Community organization membership does play a critical and enabling role in framing marginalized populations’ social and financial security, both individually and collectively, to the HIV epidemic. The study findings reaffirm that community mobilization programs are key to address structural-level issues and the reduction of vulnerabilities among sex workers. Although CO membership has increased FSWs’ acquisition of social and financial benefits, programs need to ensure through COs their active engagement in the program and continuous dialogue between the community and stakeholders. Further attention is needed to strengthen current community advocacy and engagement systems and sustain existing COs to reduce vulnerabilities and promote access to services among marginalized communities. The study also provides scope for future research using the longitudinal data to better understand the relationship of community engagement and vulnerability among FSWs.
